# Gelatin-Based Polymers Can Be Processed to Highly Resilient Biocompatible Porous Hydrogel Scaffolds for Soft Tissue Regeneration Applications

**DOI:** 10.3390/gels10110678

**Published:** 2024-10-23

**Authors:** Daniella Goder Orbach, Orna Sharabani-Yosef, Or Hadad, Meital Zilberman

**Affiliations:** 1 Department of Biomedical Engineering, Tel-Aviv University, Tel-Aviv 6997801, Israel; goderdan@mail.tau.ac.il (D.G.O.); ornashy@tauex.tau.ac.il (O.S.-Y.); 2 Department of Materials Science and Engineering, Tel-Aviv University, Tel-Aviv 6997801, Israel; orhadad21@gmail.com

**Keywords:** porous hydrogels, resilience, scaffolds, gelatin

## Abstract

Tissue regeneration relies on the mechanical properties of the surrounding environment, and it has already been shown that mechanostimulation is highly dependent on the stiffness of the native biological tissue. The main advantage of injectable hydrogels in medical applications is their ability to be delivered through minimally invasive techniques. Natural polymer-based hydrogels have been widely used in biomedical applications, due to their high biocompatibility, low immunogenicity, and similarity to soft tissues. However, the crucial combination of low stiffness with high resilience has not been achieved for natural polymers. The current study focuses on the development of novel gelatin-based injectable hydrogels for soft tissue regeneration applications, elucidating the effects of the formulation parameters on the resilience, microstructure, biocompatibility, and mechanical properties. Non-foamed hydrogels demonstrated resilience of at least 95%, while porous hydrogels maintained resilience above 90%, allowing them to withstand mechanical stresses and dynamic conditions within the body. The adjustable modulus of these hydrogels provides the necessary flexibility to mimic the mechanical properties of soft and very soft tissues, without compromising resilience. Environmental Scanning Electron Microscopy (ESEM) observations of the porous hydrogels indicated round interconnected pore structures, desired for cell migration and nutrient flow. Biocompatibility tests on fibroblasts and pre-adipocytes confirmed high biocompatibility, both directly and indirectly. In summary, structuring these new hydrogels for achieving adjustable stiffness, along with the excellent resilience and biocompatibility, is expected to enable this new technology to fit various soft tissue regeneration applications.

## 1. Introduction

Repetitive motions are common in many biological functions. For example, walking, chewing, and breathing are all based on cycles of repetitive strains on different tissues. It is therefore crucial that any material introduced into the body can mechanically withstand these cycles. Hydrogels are crosslinked three-dimensional hydrophilic structures that consist of large amounts of water [1]. They are highly suitable for biomedical applications and specifically for tissue regeneration [2]. However, hydrogels are known to have poor mechanical properties, specifically low failure strain and high stress-strain hysteresis, meaning low resilience [3].

Resilience is the ability of a material to recover its original shape, structure, and mechanical properties after undergoing deformation, such as compression or bending. A material that has high resilience would have a negligible hysteresis loop in its stress–strain curve, meaning there is very little, if any, energy dissipation [4]. It must deform elastically without a loss of energy [5].

Resilin is a common example of a naturally occurring resilient protein [6]. Natural resilin has a resilience of 92% [6], and crosslinked resilin regains its initial shape immediately after tension is released and has been shown to be 97% efficient in storing mechanical energy [5,7]. However, in compression, it was found that resilin behaves differently and can lose 27% of its energy within seconds [8]. Elastin is another natural protein which is highly resilient and can be crosslinked to form hydrogels [9,10]. Natural elastin has a resilience of 90% [10]. Although both proteins, resilin and elastin, have been widely studied for various biomedical applications, including tissue engineering, it has been shown that they are not suitable for soft tissue use, due to their high stiffness. Stiffness refers to a material’s resistance to deformation. Stiffer hydrogels are less likely to change shape when subjected to external forces like compression or tension, whereas softer hydrogels will deform more easily. Stiffness is quantified by Young’s modulus, which describes how much a material will stretch or compress under stress.

**Figure 1:**
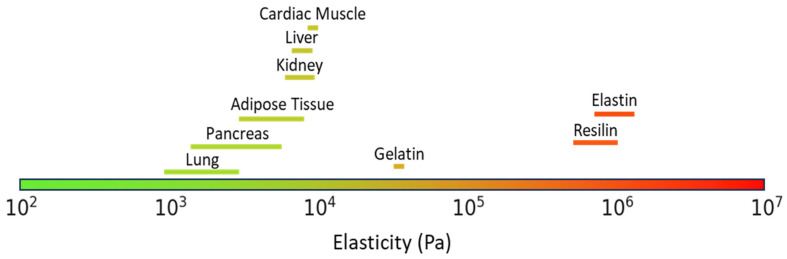
Elasticity of soft tissues and natural polymers (tissue elasticities taken from Guimarães et al. [13]).

Resilin has a Young’s modulus of 0.6–2 MPa and a compressive modulus of 2.4 MPa [11]. Elastin has a Young’s modulus in the range of 0.4–1.5 MPa [12]. Successful tissue regeneration requires the hydrogel scaffold to exhibit specific mechanical properties that are similar to those of the native tissue. In the case of soft tissues, this means having a particularly low stiffness [13,14]. Resilin and elastin both have an elastic modulus that is much higher than that of soft tissues such as adipose, lung, or brain tissues, which have all shown moduli lower than 6 kPa (Figure 1) [2,6,9,13,15].

Recent research has focused on finding a solution for the high energy loss during loading and unloading of hydrogels, and has focused on synthetic polymers. Liu et al. [4] synthesized a tough and resilient crosslinked acrylamide-based hydrogel which incorporates helical peptide chains that function as springs, increasing resilience. Poly (ethylene-glycol) and polydimethylsiloxane hydrogels, synthesized by Cui et al. [3], exhibited high resilience as well as elasticity that is comparable to resilin. However, synthetic hydrogels are not ideal for biomedical applications due to their acidic degradation products that may lead to a range of adverse effects [16]. Biocompatibility is much higher in natural polymers, such as gelatin and alginate. Additionally, their structure is inherently similar to the native extracellular matrix, which enables and improves cell adhesion when compared to synthetic polymers [2].

Gelatin is a water-soluble derivative of collagen, the most commonly occurring protein in the extracellular matrix, and as such, it has been widely studied for biomedical applications. It is biocompatible, biodegradable, and non-immunogenic [17,18]. Fish gelatin, which is used in this study, is composed mainly of the imino acid glycine (approximately 30%), and has a high concentration of proline and alanine as well, but because gelatin is obtained by degradation of collagen, it has a heterogenic and complex structure [19,20]. Fish gelatin has lower rheological properties than mammalian gelatin, which decreases the gelation temperature to below room temperature [21].

Gelatin has been used in transdermal [22], ocular [23,24], and buccal [25] drug delivery systems. It has also been studied for use in cartilage [26] and cardiac tissue [27] reconstruction, and generally as a tissue regeneration scaffold [28,29]. At low strains, gelatin has a resilience of 90%; however, at 40% strain, gelatin hydrogels fail [3]. To improve its resilience, several studies investigated combinations of gelatin with synthetic polymers. For example, Gan et al. [30] increased the resilience of commonly studied gelatin methacryloyl hydrogels by incorporating oligomers of dopamine methacrylate, and Chen et al. reported a gelatin-[tris(hydroxymethyl)methyl] acrylamide hydrogel for sealing gastric perforations that can be stretched or compressed and regains its original shape [30].

To avoid the disadvantages of synthetic polymers, we chose to base our hydrogel solely on natural polymers. The main component of our hydrogels is gelatin, and sodium alginate is added in relatively low concentrations for the purpose of controlling the scaffold’s physical properties, such as gelation time and swelling degree [31]. Sodium alginate is a natural polysaccharide derived from brown algae. It is a linear, unbranched copolymer composed of covalently linked blocks of (1,4)-linked β-D-mannuronic acid and α-L-guluronic acid and has a high concentration of carboxylic groups. Its properties depend on its molecular weight and the mannuronic/guluronic ratio [32]. Alginate is commonly used in biomedical applications, due to its high biocompatibility, biodegradability, and versatility [33]. Tegaderm™ wound dressing is a good example for an alginate-based product developed to provide a moist environment for wound healing [34]. Alginate has also been used in drug delivery [35] and tissue engineering applications [36,37,38].

Natural polymers are versatile and extensively studied for various biomedical applications, but they do not have the mechanical properties necessary for withstanding cyclic loads. They are neither strong nor resilient. Processing them into structures with desired mechanical properties, to fit specific tissue regeneration applications, is still an unmet need. Crosslinking has been shown to improve mechanical properties [2]. The crosslinking agent chosen for the current study is N-ethyl-N-(3-dimethylaminopropyl) carbodiimide (EDC). EDC is a zero-length crosslinker that has been reported to be less toxic than other conventional crosslinking agents for protein crosslinking, such as formaldehyde and glutaraldehyde [39]. EDC activates the carboxylic groups in the gelatin and alginate, which form a highly reactive o-iso-acylurea. It then undergoes a nucleophilic attack by a primary amino group form the gelatin or alginate, and forms an amide bond along with a urea molecule which is released as a byproduct. This crosslinking reaction creates a hydrogel [40,41]. Even though crosslinking is a known and common method to improve hydrogel mechanical properties, a high resilience combined with low stiffness has yet to be achieved using natural polymers.

Hence, the current study focuses on development and characterization of natural polymer-based resilient hydrogels that are able to almost completely regain their original shape after repetitive compression and release cycles, and withstand 90% compression without failing. In addition, they combine low stiffness with high resilience, making them prime candidates for soft tissue regeneration applications. Their resilience behavior and structural features are described in this manuscript. Additionally, their biocompatibility was evaluated directly and indirectly and is presented here.

## 2. Results and Discussion

**Figure 2:**
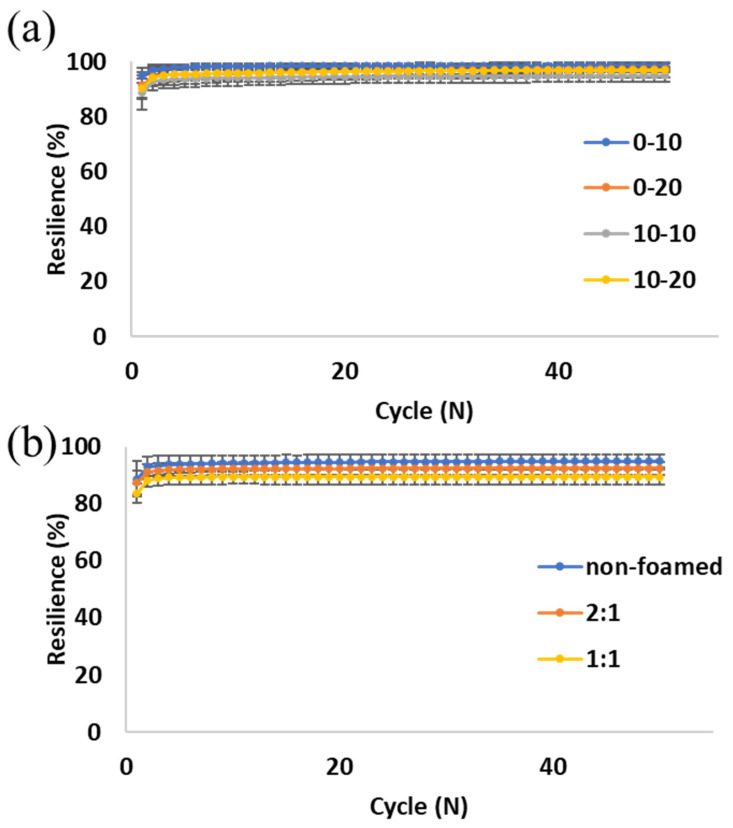
Hydrogel resilience. (**a**) Different formulations of non-foamed hydrogels (alginate-EDC concentrations, mg/mL); (**b**) Different foaming ratios (polymer:air) of 10-10 alginate-EDC hydrogel. Error bars indicate one standard deviation, n ≥ 5.

Resilience can be measured by the difference in energy, stored and released from the hydrogel during a loading-unloading cycle (Equation (1)). Figure 2 shows resilience as a measure of the recovered energy upon the removal of the load, divided by the total energy during compression of the sample [5]. All formulations in all foaming ratios exhibited very high resilience during all 50 loading cycles. Figure 2a shows a comparison between various formulations. The inclusion of alginate slightly lowers the resilience of the hydrogel, but this effect is counteracted by the addition of a higher concentration of EDC (20 instead of 10 mg/mL). It is known that EDC crosslinks between gelatin and gelatin and between gelatin and alginate molecules [42,43,44]. Thus, when adding alginate molecules to gelatin, gelatin–alginate bonds are created instead of some of the gelatin–gelatin bonds. This may increase the chain entanglements and, as a result, decrease the chain flexibility and thus resilience as well. Additionally, it is hypothesized that when a relatively high concentration of alginate is added to gelatin, more alginate–gelatin bonds are created instead of gelatin–gelatin bonds that could occur within the same molecule. Because in this case, two or more molecules are connected, longer polymer chains are likely created, probably increasing intramolecular friction and therefore lowering resilience [45]. However, since the alginate concentrations in our formulations are significantly lower compared to the gelatin concentration, they only slightly affect the resilience, which remains very high (above 95%) even after 50 cycles.

**Figure 3:**
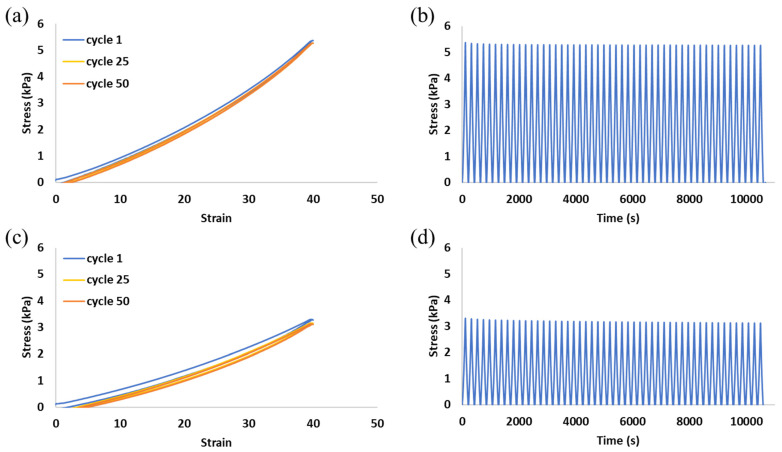
Representative stress–strain curves of the first, middle, and last loading and unloading cycles (**a**,**c**), and stress of the hydrogels during 50 consecutive cycles (**b**,**d**) of 10-10 mg/mL alginate-EDC non-foamed formulations and 1:1 polymer:air foaming ratio, respectively.

After the first cycle, which can be regarded as preconditioning, all formulations exhibit a resilience of over 90% throughout the experiment, except for the 1:1 foaming ratio, which starts at 89% (Figure 2b). This is likely caused by the relatively high porosity of this sample. As the sample compresses, the air pockets collapse, and when the load is removed, they regain their original size. An increase in porosity leads to a decrease in the polymer content, i.e., there is less material to resist deformations and maintain the sample shape. Although our 1:1 foamed hydrogels are highly porous, they barely lose any energy during the loading and unloading cycles and thus remain resilient, as can be seen in Figure 3.

Figure 3 presents two examples of the hysteresis loops on the stress–strain curve during the first, middle, and last loading–unloading cycle (Figure 3a,c) and the stress over time during 50 loading–unloading cycles (Figure 3b,d). The results of the other studied formulations are similar to the presented results and therefore are not shown here. A very small hysteresis loop is observed in all formulations, and it remains nearly unchanged for the entire set of compression and release cycles. The elasticity of the samples can be seen in the stress over time graphs, as the stress applied to the samples remains consistent during all cycles, indicating the retention of the samples’ size and shape.

**Figure 4:**
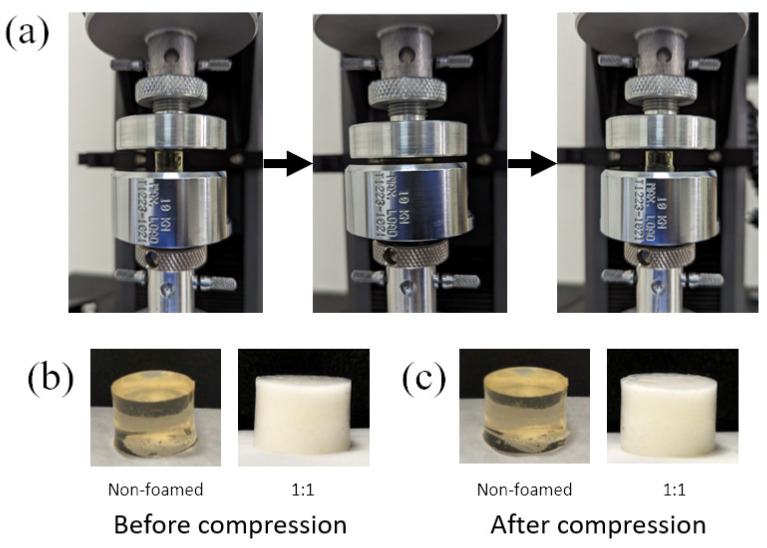
10-10 mg/mL alginate-EDC (**a**) non-foamed hydrogel sample: before, during, and after (from left to right) compression to a strain of 90% and release; (**b**,**c**) non-foamed and 1:1 polymer:air foamed sample before and after compression, respectively.

Following the excellent resilience that the hydrogels exhibited at 40% strain, hydrogel samples were loaded to a strain of 90% or a force of 9N. The samples did not fail; they were compressed until the maximum strain (or force) was reached and they regained their initial shape after release (Figure 4a). Close-ups of a non-foamed and 1:1 polymer-to-air foamed hydrogel cylinders before (Figure 4b) and after (Figure 4c) compression are also presented. The 2:1 foaming ratio is not presented because of its visual similarity to the 1:1 sample. The maximum change in dimensions after compression was observed in the 0–10 mg/mL alginate-EDC 1:1 foamed sample, where a decrease in height of 4.1% had occurred. Other samples lost between 1.3 and 3.6% of their height due to the compressive force. It is therefore clear that there are no major changes to the macrostructure of the hydrogel after the application of large strains.

**Figure 5:**
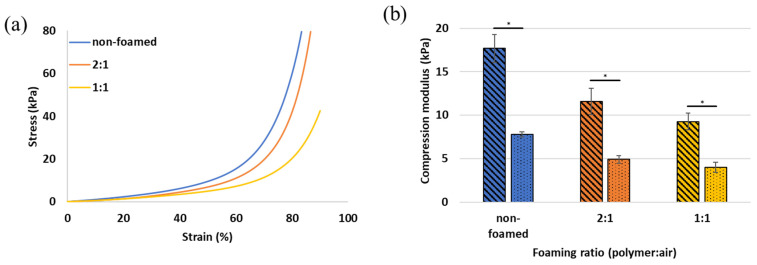
(**a**) Representative compressive stress–strain curves of 10-10 mg/mL alginate-EDC hydrogel, non-foamed or foamed with a polymer:air ratio of 2:1 and 1:1. Graphs go up to either 90% strain or a force of 9N; (**b**) the compression modulus of the hydrogels before swelling (striped) and after swelling (dotted); *p*-value < 0.5 is represented by *.

Stress–strain curves of 10-10 mg/mL alginate-EDC with various foaming ratios are presented in Figure 5a, and the resulting compression moduli before and after swelling are presented in Figure 5b. Similar effects of the various foaming ratios on the stress–strain curves and of the swelling on the compression modulus were observed in the 10-20 mg/mL alginate-EDC formulations. An increase in porosity results in a lower compression modulus, as seen in both parts of Figure 5. During loading, part of the pores (which are actually air pockets) collapse; they do not resist the compression as the hydrogel itself does, and as a result the scaffold becomes more flexible. Additionally, when the polymer undergoes swelling, as expected in the aqueous in vivo environment, its compression modulus decreases even further, bringing the mechanical properties to the range of very soft tissues. It should be noted that swelling was performed in water, whereas in vivo the scaffold would be surrounded by complex biological fluids, which may vary depending on the implantation site. Therefore, in vivo experiments should be performed to assess the change in hydrogel properties over time, caused by the presence of the hydrogel in specific biological environments.

The combination of percent resilience (Figure 2b), the stress–strain curves (Figure 5a), and the compression moduli (Figure 5b) makes it apparent that the slightly lower resilience of the 1:1 foaming ratio is accompanied by a decrease in modulus. This is caused by the higher pore percentage of the 1:1 polymer:air foaming ratio. It is important to note that even small polymer-to-air ratio samples, which result from low polymer content, are slightly affected when the hydrogel is subjected to compression forces, and this has some effects on the resilience and the elasticity of the scaffold. Therefore, the decrease in resilience in such cases is negligible, making this platform tailorable for various applications that require compliance as well as resilience. For example, lung tissue which has a relatively low Young’s modulus (1–6 kPa) should withstand the repetitive breathing motions [46], and adipose tissue undergoes constant compression and extension throughout the body, with a range of low moduli, depending on the type of fat [47].

**Figure 6:**
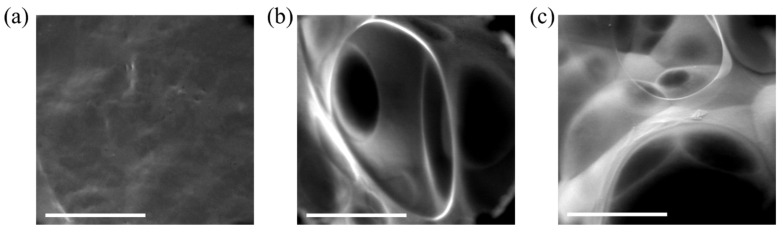
ESEM images of 10-10 mg/mL alginate-EDC hydrogel: (**a**) non-foamed; (**b**) foamed with a polymer:air ratio of 2:1; and (**c**) foamed with a 1:1 polymer:air ratio. Scale bar: 200 µm.

Environmental Scanning Electron Microscope (ESEM) images of 10-10 mg/mL alginate-EDC non-foamed and foamed hydrogels with two foaming ratios are presented in Figure 6a–c, respectively. Images of 10-20 mg/mL alginate-EDC hydrogels were visually similar and are therefore not presented. Non-foamed samples had no pores, as expected. In foamed samples, the porosity was homogenous throughout the sample. Pores were found to be round and interconnected, which is crucial in tissue engineering scaffolds. It has been previously shown that in order for cells to migrate and adhere to an implanted scaffold, a porous structure with connected pores is needed [48,49] to enable nutrient influx as well as removal of waste products, which are both important for tissue ingrowth. These pores affect the compression modulus of the hydrogels, as seen in Figure 5, but do not affect the resilience as seen in Figure 3. Our hydrogels can be easily made to be non-porous or porous while maintaining the high resilience which makes their uses versatile.

To ensure that cells can thrive in and around the scaffold, cell viability tests were performed using the AlamarBlue™ assay. The results are presented in Table 1. According to ISO 10993 [50], for a material to be considered non-cytotoxic, it has to allow for cell viability of over 70% after 24 and 48 h. All hydrogel formulations exhibited excellent viability, most over 100% when compared to a control well, consisting of cells seeded in fresh medium.

The high biocompatibility of the EDC crosslinked gelatin–alginate hydrogels results from the fact that EDC is a zero-length crosslinker, which means that after creating gelatin-gelatin and gelatin–alginate bonds, it exits the reaction as a urea byproduct. As long as the urea concentrations are not high, they do not induce cytotoxic effects. Since EDC itself is not included in the final crosslinked hydrogel, cytotoxic effects are also not expected upon degradation of the hydrogel.

**Figure 7:**
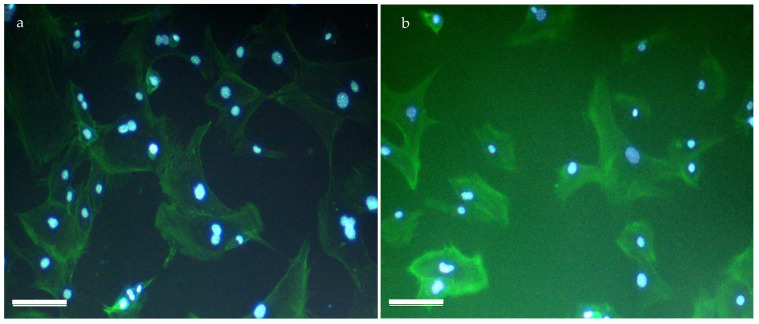
DAPI and Phalloidin staining 48 h after seeding on the scaffold; 100× magnification of an overlap of DAPI and Phalloidin staining in (**a**) 1:1 polymer:air and (**b**) 2:1 polymer:air foamed hydrogels. Scale bar: 100 μm.

The hydrogel’s biocompatibility was further evaluated using a direct approach. Mouse pre-adipocytes were seeded on the hydrogel, and after 48 h of incubation, both the scaffold and cells were stained using DAPI and phalloidin. Figure 7 demonstrates successful cellular adhesion to both the 1:1 and 2:1 polymer:air foamed samples. The clear overlay of F-actin filaments (green) and presence of nuclei (blue) indicate a spread-out, healthy cell morphology.

**Figure 8:**
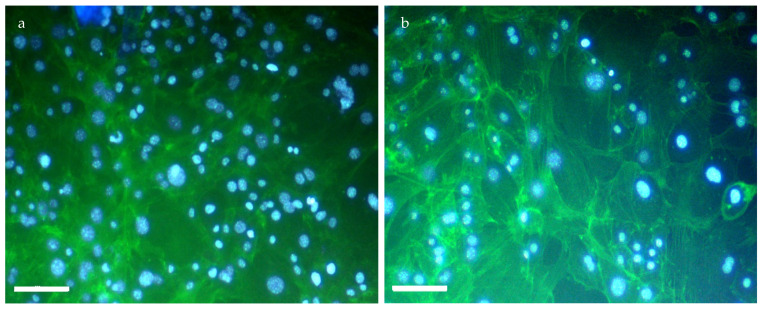
Cells at the bottom of the wells where the seeded hydrogel was incubated, after removing the scaffold. Overlap of 100× magnification after DAPI and Phalloidin staining of (**a**) 1:1 polymer:air and (**b**) 2:1 polymer:air foamed samples. Scale bar: 100 μm.

To assess long-term biocompatibility, and whether the cells can migrate through the scaffold’s pores, cells were seeded on top of the scaffold and cultured for 14 days. After removing the scaffold, the contents of the well were stained with DAPI and phalloidin (Figure 8) to see if cells were present below the scaffold. Because the hydrogel covered the entire surface of the well, cells found on the bottom means that they have successfully migrated through the hydrogel. The abundant presence of both blue (DAPI) and green (phalloidin) signals indicates several positive outcomes. First, the strong signals confirm cell viability and proliferation after 14 days, demonstrating the scaffold’s lack of cytotoxicity and good biocompatibility. Second, the presence of stained cells at the well’s bottom (opposite the initial seeding location) suggests the cells successfully migrated through the scaffold’s pores. This highlights the scaffold’s potential to support cell infiltration.

The combination of the indirect cytotoxicity tests (Table 1) and successful direct cell seeding experiments (Figure 7 and Figure 8) demonstrates the hydrogel’s excellent biocompatibility. This biocompatibility allows cells to adhere, spread, and potentially migrate within the scaffold, indicating their viability and functionality.

Future research should contain in vivo experiments, testing not only cellular viability on and around the scaffold, but also infiltration and differentiation. Scaffold resilience is expected to withstand repetitive mechanical loads that will allow for mechanostimulation to occur, leading to cellular differentiation, and ultimately to successful tissue regeneration.

## 3. Conclusions

Our research focuses on the development of novel gelatin-based biodegradable, injectable hydrogels for soft tissue regeneration, and the study of their resilience, biocompatibility, and mechanical properties. These hydrogels demonstrated exceptional resilience during repeated loading–unloading cycles, with nearly consistent performance in all studied formulations.

The incorporation of alginate as a secondary natural component resulted in some decrease in resilience due to increased polymeric chain friction. Despite this, the resilience remained high, and the hydrogels maintained their shape and functionality under repetitive 40% strain conditions. The various polymer-to-air ratios resulted in soft hydrogels with tunable mechanical properties.

The unique combination of low modulus and high resilience of these hydrogels, which is desired for soft tissue regeneration application, is achieved for the first time in natural polymer-based technologies. It is achieved due to the inherent properties of the chosen polymers, the specific formulations which contained high gelatin concentrations, low alginate concentrations, and the partial crosslinking.

ESEM imaging demonstrated foamed hydrogels with round interconnected pores, which enhanced their suitability for tissue engineering applications by supporting cell migration and nutrient flow. The biocompatibility of these new scaffolds was tested on fibroblasts and pre-adipocytes, which showed high biocompatibility both directly and indirectly.

This versatile material can be tailored to either porous or non-porous forms while retaining high resilience, making it a promising candidate for a range of biomedical applications requiring both flexibility and mechanical durability. This mechanical performance, together with their high biocompatibility, and being highly porous, makes our new hydrogels prime candidates for soft tissue regeneration applications.

## 4. Materials and Methods

Cold-water fish gelatin (G7041, Sigma-Aldrich) and alginic acid sodium salt (A1112, Sigma-Aldrich) were used to prepare a polymeric solution that was crosslinked using N-(3-dimethylaminopropyl)-N’-ethylcarbodiimide hydrochloride (EDC) (E7750, Sigma-Aldrich).

### 4.1. Hydrogel Preparation

Preparation of the hydrogels was based on the preparation and mixing of two solutions. Double distilled water was heated to 60 °C, then gelatin and alginate were added and mixed until dissolved, to prepare a polymeric solution. EDC was added to double-distilled water to prepare the crosslinking solution. The two solutions were homogeneously mixed using a commercial double syringe, fitted with a static mixer tip (Mixpac™, L-system 2.5 mL, 4:1 volume ratio, purchased from Sulzer Mixpac, AG, Haag, Switzerland).

Porous hydrogels were created by mixing the polymeric solution with air prior to loading the double syringe. The polymeric solution was loaded into a regular syringe, and a specific volume of air was loaded into a second syringe. By vigorously mixing the two using a 3-way stopcock, a foamed polymer–air solution was formed which was then loaded into the double-syringe. The contents of the double-syringe were then immediately cast into molds to avoid the polymer–air mixture from separating. Gelation times of the hydrogels vary between 5 and 30 s [51], allowing them to be cast into the molds before they crosslink and become a gel. In a previous study [52], the rheological properties of similar hydrogels were examined, demonstrating the injectability of the gelatin-alginate-EDC hydrogels reported in this paper, and in a thesis of one of the research group’s graduates, bubble size and dispersion throughout the sample were found to be consistent within each foaming ratio [53].

A series of hydrogels were prepared, all based on a gelatin concentration of 200 mg/mL, with various concentrations of alginate and EDC, and foaming ratios of 1:1 and 2:1 volumetric polymer: air ratio, as well as non-foamed. The volumetric foaming ratio was determined by the volumes of polymeric solution and air that were mixed prior to loading the double syringe. The studied formulations are presented in Table 2.

### 4.2. Mechanical Properties

Mechanical tests were conducted using a 5500 Instron Universal Testing Machine (model 5944) fitted with a 10 N load cell. Cylindrical samples, measuring 12 mm in diameter and 8 mm in height, were prepared by extruding the solutions through a double syringe into silicone molds. Samples were kept in a covered petri dish at room temperature for one hour prior to testing.

#### 4.2.1. Resilience

The resilience of the hydrogels was evaluated by subjecting the samples to 50 compression cycles, applying 40% strain, and releasing them to their original height at a rate of 5 mm/min. Resilience was calculated using the following equation [3]:

(1) $$\mathrm{Resilience}\left(\%\right)=\frac{L-U}{L}\times 100$$

L represents the area under the loading stress–strain curve, signifying the energy required for deformation. U represents the area under the unloading stress–strain curve, signifying the release of stored energy. The difference L − U forms the hysteresis loop, indicating energy dissipation. At least five repetitions were performed for each formulation.

#### 4.2.2. Compression

Compression after water uptake was tested by performing the compression method described above on samples after incubation in water, at 37 °C, for 24 h. At least five repetitions were performed for each formulation.

### 4.3. Microstructure

The morphological analysis of the samples was conducted using Environmental Scanning Electron Microscopy (Quanta 200 FEG ESEM). The ESEM was operated in low vacuum mode, at a high voltage of 20 kV, with a working distance (WD) of 5.9 mm. These parameters were chosen to optimize the resolution and contrast of the images while preserving the integrity of the hydrated samples.

### 4.4. Cell Viability

In vitro indirect cell viability tests were carried out on primary human neonatal fibroblasts (Rambam Medical Center, Haifa, Israel) and mouse 3T3L pre-adipocytes (American Type Culture Collection, Manassas, VA, USA) using an indirect method in accordance with ISO 10993:12 (parts 5 and 12) for the biological evaluation of medical devices [50]. Non-foamed hydrogels were selected for the study, as using foamed hydrogels, according to the extract liquid volume guidelines for indirect cytotoxicity tests (Table 1, part 12 of ISO 10993 [50]), would have required a smaller hydrogel weight per mL of extract medium. This approach was chosen to test the maximum risk condition. After 24 h of incubation, the cell culture medium was replaced with the hydrogel extract, and the cells were incubated for an additional 24 or 48 h before their viability was assessed using the AlamarBlue™ assay (Invitrogen™, Rhenium, Modi’in, Israel).

Direct cell viability tests were performed by seeding mouse pre-adipocytes directly on the scaffold. Cell seeding and staining were done using Dulbecco’s Phosphate Buffered Saline (PBS, Sartorius, Biological Industries, Beit Ha’emek, Israel), Trypsin A (Sigma-Aldrich, Rehovot, Israel), Trypan blue solution (0.4%, Sigma-Aldrich, Rehovot, Israel), Triton-X100 solution (Sigma-Aldrich, Rehovot, Israel), formaldehyde solution (Sigma-Aldrich, Rehovot, Israel), mounting medium with DAPI (4′,6-Diamidine-2′-phenylindole dihydrochloride, Abcam, Zotal Ltd., Tel Aviv, Israel) and a Phalloidin—iFluor 488 reagent (Abcam, Zotal Ltd., Israel).

Mouse pre-adipocyte cells were cultured with an MEM medium solution containing MEM, 10% fetal calf serum (Rhenium, Modi’in, Israel) 2mM L-Glutamine and 0.1% penicillin/streptomycin (Biological Industries, Beit Ha’emek, Israel), and incubated in a 37 °C, 5% CO_2_ environment. The cells were suspended in medium and seeded on top of the hydrogel. In addition, control samples of only cells and unseeded hydrogel were prepared.

The hydrogel was inspected under an optical microscope (Nikon Eclipse Ts2), and three methods of cell staining were tested: Trypan blue cell staining for viability, and DAPI and phalloidin fluorescence staining for cell nucleus and structure, respectively. DAPI and phalloidin staining were observed using a fluorescence microscope (Zeiss, Oberkochen, Germany, Axiovert 40 CFL), using the appropriate filters (Ex/Em = 358/461 nm and 493/517 nm, respectively) without moving the scaffold to show the overlap of the nuclei and actin network. Images were processed and overlapped with ImageJ software 1.53.

### 4.5. Statistical Analysis

All data were processed using Microsoft Excel. Statistical analysis was performed using ANOVA (Tukey Kramer post hoc) via IBM SPSS (v. 27). A *p*-value of <0.05 is marked with an * in figures.

## Figures and Tables

**Table 1:** Fibroblast and pre-adipocyte viability after 24/48 h in hydrogel extracts, as a percentage of viability in control wells without hydrogel. Viability above 70% is considered by the FDA to be non-cytotoxic.

Formulation (mg/mL)	Fibroblasts	Pre-Adipocytes
10-10 Alginate-EDC	104±11/105±4	94±10/101±9
10-20 Alginate-EDC	101±13/79±4	102±15/96±9

**Table 2:** The formulations used in the study. All formulations are based on 200 mg/mL gelatin.

Alginate Concentration (mg/mL)	EDC Concentration (mg/mL)	Foaming Ratio (Polymer:Air)
0	10, 20	Non-foamed 2:1 1:1
10	10, 20	Non-foamed 2:1 1:1

## Data Availability

Dataset available on request from the authors.
